# Analysis of large deletions in *BRCA1*, *BRCA2 *and *PALB2 *genes in Finnish breast and ovarian cancer families

**DOI:** 10.1186/1471-2407-8-146

**Published:** 2008-05-26

**Authors:** Katri Pylkäs, Hannele Erkko, Jenni Nikkilä, Szilvia Sólyom, Robert Winqvist

**Affiliations:** 1 Laboratory of Cancer Genetics, Oulu University Hospital and University of Oulu/Biocenter Oulu, Oulu, Finland

## Abstract

**Background:**

*BRCA1 *and *BRCA2 *are the two most important genes associated with familial breast and ovarian cancer susceptibility. In addition, *PALB2 *has recently been identified as a breast cancer susceptibility gene in several populations. Here we have evaluated whether large genomic rearrangement in these genes could explain some of Finnish breast and/or ovarian cancer families.

**Methods:**

Altogether 61 index patients of Northern Finnish breast and/or ovarian cancer families were analyzed by Multiplex ligation-dependent probe amplification (MLPA) method in order to identify exon deletions and duplications in *BRCA1*, *BRCA2 *and *PALB2*. The families have been comprehensively screened for germline mutation in these genes by conventional methods of mutation analysis and were found negative.

**Results:**

We identified one large deletion in *BRCA1*, deleting the most part of the gene (exon 1A-13) in one family with family history of ovarian cancer. No large genomic rearrangements were identified in either *BRCA2 *or *PALB2*.

**Conclusion:**

In Finland, women eligible for *BRCA1 *or *BRCA2 *mutation screening, when found negative, could benefit from screening for large genomic rearrangements at least in *BRCA1*. On the contrary, the genomic rearrangements in *PALB2 *seem not to contribute to the hereditary breast cancer susceptibility.

## Background

Breast cancer is the most frequently occurring malignancy in women. *BRCA1 *and *BRCA2 *are the two major susceptibility genes, accounting for varying fraction of familial breast and ovarian cancer cases in different populations. In Finland, mutations in these genes explain approximately 20% of breast and ovarian cancer families [1,2]. Most of the alterations identified in *BRCA1 *and *BRCA2 *are point mutations and small insertions/deletions, but increasing number of large genomic rearrangements in both genes have been identified in different populations [3-6]. Rearrangements have been described throughout the genes, and majority of them are unique and introduce a premature termination codon in the reading frame [3,6]. The proportion of *BRCA1 *and *BRCA2 *mutations due to genomic rearrangements is not expected to vary markedly in different populations, although there might be accumulation of certain mutations due to a founder effect.

Previous studies performed in the Finnish population have not observed large genomic rearrangements in *BRCA1 *or *BRCA2 *[7-9]. However, in the previous studies either the method used (Southern blotting analysis on part of these genes) has not allowed sensitive testing [7], the study has concentrated only on male breast cancer cases [9] or the analyzed samples were derived from a geographically restricted area [8]. In Finland, the difference in geographical distribution has been reported for several cancer susceptibility alleles, including *BRCA1*, *BRCA2*, *ATM *and *RAD50 *mutations [2,10-12], which is the result of strong founder effect and population history. The settlement was restricted to the coastal areas during the 15^th ^century, and it was not until the 17^th ^century that the vast inland regions were gradually inhabited by a relatively small number of individuals, resulting in several regionally occurring founder mutations [2]. Consequently, large genomic rearrangements in *BRCA1 *and *BRCA2 *might still be at least partly responsible for the hereditary predisposition to breast and ovarian cancer in Finland.

*PALB2 *was recently identified as a breast cancer susceptibility gene [13,14] and mutations in it have since been reported in other populations [15-17]. *PALB2 *encodes a protein that binds to BRCA2 and this interaction is crucial for certain BRCA2 DNA damage response and tumor suppression functions [18]. The breast cancer associated mutations identified in *PALB2 *are expected to be deleterious, and all result in protein truncations. The risk estimates for *PALB2 *mutations have ranged from two- to fourfold, although some *PALB2 *mutations have been suggested to have higher penetrance [13-15]. Here, we wanted to investigate whether large genomic rearrangements in *PALB2 *could also explain some of the Finnish breast and/or ovarian cancer families.

In the current study we have used Multiplex ligation-dependent probe amplification (MLPA) in order to identify exon deletions and duplications in the *BRCA1*, *BRCA2 *and *PALB2 *genes. MLPA has been proven to be very useful in detecting copy number changes in genomic sequences [19]. The families selected to this study have been comprehensively screened for germline mutation in these genes by conventional methods of mutation analysis [2,13] and were found negative.

## Methods

### Breast and/or ovarian cancer families

Altogether 61 index patients of Northern Finnish breast and/or ovarian cancer families were selected for the study. These families have been screened for germline mutations in *BRCA1*, *BRCA2 *and *PALB2 *and were found negative [2,13]. 41 of the families had three or more cases of breast and/or ovarian cancer in first- or second-degree relatives, and 11 families had two cases of breast and/or ovarian cancer in first- or second-degree relatives, of which at least one with early disease onset (≤ 35 years), bilateral disease or multiple primary tumors. Four families had one case of breast and ovarian cancer each, three families had two breast cancer cases, of which one diagnosed at young age ≤ 43 years, and two families showed one breast cancer case (≤ 40 years) with multiple primary tumors. The affected index cases of these families were analyzed, and the patient with youngest age at diagnosis or the one with multiple tumors was selected as an index. The study has been approved by the Ethical Board of the Northern Ostrobothnia Health Care District and the Finnish Ministry of Social Affairs and Health. All patients provided informed consent to participate in this study.

### MLPA and data analysis

The SALSA MLPA kits for *BRCA1 *(primary screening kit P002B and confirmation kit P087), *BRCA2 *(P045B) and *PALB2 *(P057) kits (MRC-Holland, Netherlands) were used according to the manufacturer's instructions. After PCR amplification with IRD800 labeled primers the samples were analyzed with Li-Cor IR^2 ^4200-S DNA Analysis system (Li-Cor Inc., Lincoln, NE) and Gene Profiler 4.05 analysis program (Scanalytics, Inc., Fairfax, VA). For *BRCA1 *analysis the used deletion control had deletions in exons 1A-2, for *BRCA2 *analysis the control sample showed constant 50% reduction in band intensity resulting from a SNP locating three base pairs from the ligation site, and for *PALB2 *analysis the used control had heterozygous deletions in exons 1–3 and 5–10 in addition to homozygous deletion of exon 4.

The information regarding the integrated density of each band received from GeneProfiler was analyzed by MLPA spreadsheets (National Genetics Reference Laboratory) in Excel Software according to the instructions. Dosage quotients 0.35–0.65 were considered deleted and dosage quotients 1.35–1.65 duplicated, and samples with quality value (standard deviation of the control ligation products) exceeding 0.1 were rejected. For the DNA sample positive for a genomic rearrangement, analysis was repeated using an independent sample in an independent assay.

## Results

**Figure 1:**
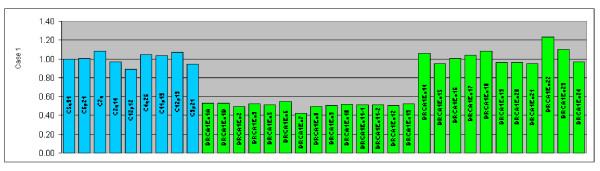
**MLPA analysis of a patient with family history of ovarian cancer.** A large genomic deletion of exons 1A to 13 in *BRCA1 *was found. Y-axis dosage quotients, X-axis used control probes (blue) and individual *BRCA1 *exons (green).

**Figure 2:**
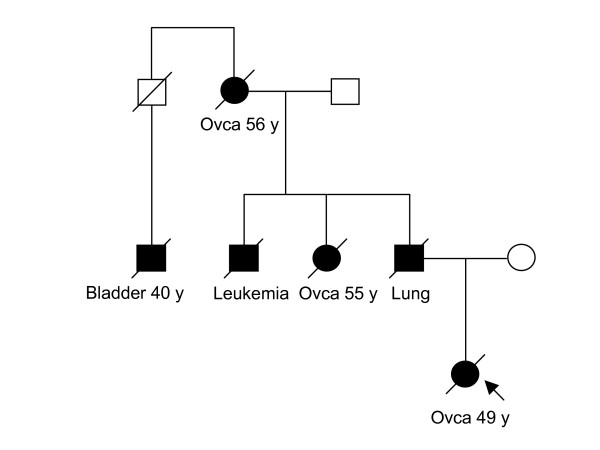
**Family exhibiting exon 1A-13 deletion in *BRCA1*.** Index case is shown with an arrow.

We have analyzed a total of 61 index patients for large genomic rearrangements in *BRCA1*, *BRCA2 *and *PALB2 *genes by MLPA. In *BRCA2 *and *PALB2 *no deletions or duplications were observed. We did, however, observe one large deletion in *BRCA1 *(exon 1A-13) (Figure 1), deleting the most part of the gene, including the promoter region. This deletion spans over 43 kb. The initial observation with P002B kit was subsequently confirmed by the *BRCA1 *confirmation kit P087. The patient carrying the deletion allele was diagnosed with ovarian cancer at the age of 49 years. The family had strong history of cancer, and there were altogether three cases of ovarian cancer (Figure 2). DNA was not available for testing from any other family members.

## Discussion

Germline mutations in *BRCA1 *and *BRCA2 *cause an increased lifetime risk for breast and ovarian cancer [20]. In addition, *PALB2 *has recently been identified as a breast cancer susceptibility gene in several populations, but its association with increased risk for ovarian cancer has not been established [13-17]. Although *BRCA1 *and *BRCA2 *rearrangements have previously been studied in the Finnish population, none have so far been reported [7-9]. The earlier studies have, however, been concentrating on families deriving from geographically restricted area or only on male breast cancer patients, or have used the less sensitive Southern blotting method. Therefore, a comprehensive analysis of genomic rearrangements in the *BRCA1 *and *BRCA2 *genes in the Northern Finnish patient cohort was needed. For *PALB2 *this is the first study designed to detect large genomic rearrangements in the Finnish population.

The index cases of 61 families included in this study were analyzed for rearrangements in the three genes by MLPA, and in one family we identified a large deletion in *BRCA1*. The observed deletion removes most of the gene including the promoter [21], thereby preventing the transcription of *BRCA1*. The mutation positive patient displayed a family history of ovarian cancer, which has been shown to increase the likelihood of finding a *BRCA1 *mutation in a family [22]. Deletions that remove the *BRCA1 *promoter have previously been described, but the earlier studies have not associated these changes with any particular phenotype [23-26]. Our result provide the first evidence that, like in many other studied populations, large genomic changes in *BRCA1 *do also exist in Finland. However, these mutations seem to be rare, if not unique, as this deletion was seen in only one out of 61 families.

To date, only three different *BRCA1 *mutations have been identified in Northern Finnish breast and/or ovarian cancer families. Two of these mutations, 3745delT and 4216-2ntA>G, represent recurrent Finnish founder mutations [1,2,27], accounting for three mutation positive families each [2]. The third *BRCA1 *mutation is the currently identified large deletion of exons 1A-13, which represents 14.3% (1/7) of the identified Northern Finnish *BRCA1 *positive families. Even though the deletion allele was observed only in one out 61 currently analyzed families, our results suggests that women eligible for *BRCA1 *or *BRCA2 *mutation screening, when found negative, could benefit from screening for large genomic rearrangements, at least in *BRCA1*. Additional work is still needed in order to determine the prevalence of *BRCA2 *rearrangements in Finland, as the current study was based only on relatively small number of families.

Despite the identification of one large genomic deletion in *BRCA1*, our results support the previous conclusions that the genomic rearrangements in *BRCA1 *and *BRCA2 *are not a major cause for increased breast cancer susceptibility in Finland, and that the previously reported Finnish founder mutations represent the majority of *BRCA1 *and *BRCA2 *positive families [1,2,7-9,27]. No exon deletions or duplications of the *PALB2 *gene were identified in the studied index cases of 61 families. This suggests that genomic rearrangements in *PALB2 *are very rare, which has also been indicated by a previous study [16]. At least in the Finnish population the major breast cancer associated aberration in *PALB2 *appears to be the previously reported founder truncation mutation [13].

## Conclusion

In Finland, women eligible for *BRCA1 *or *BRCA2 *mutation screening, when found negative, could benefit from screening for large genomic rearrangements at least in *BRCA1*. In contrast, the genomic rearrangements in *PALB2 *seem not to contribute to the hereditary breast cancer susceptibility in Finland.

## Competing interests

The authors declare that they have no competing interests.

## Authors' contributions

KP carried out the MLPA and data analysis, and drafted the manuscript. HE, JN and SS helped to draft the manuscript. RW participated in study design and in drafting the manuscript. All authors read and approved the final manuscript.

## Pre-publication history

The pre-publication history for this paper can be accessed here:


